# Ten Years’ Experience with the CYP2D6 Activity Score: A Perspective on Future Investigations to Improve Clinical Predictions for Precision Therapeutics

**DOI:** 10.3390/jpm8020015

**Published:** 2018-04-17

**Authors:** Andrea Gaedigk, Jean C. Dinh, Hyunyoung Jeong, Bhagwat Prasad, J. Steven Leeder

**Affiliations:** 1 Division of Clinical Pharmacology, Toxicology & Therapeutic Innovation, Children’s Mercy Kansas City, 2401 Gillham Rd, Kanas City, MO 64108, USA; jdinh@cmh.edu (J.C.D.); sleeder@cmh.edu (J.S.L.); 2 School of Medicine, University of Missouri-Kansas City, Kansas City, MO 64108, USA; 3 College of Pharmacy, University of Chicago, IL 60607, USA; yjeong@uic.edu; 4 Department of Pharmaceutics, University of Washington, Seattle, WA 98195, USA; bhagwat@uw.edu

**Keywords:** CYP2D6, activity score, dextromethorphan, inter-individual variability

## Abstract

The seminal paper on the CYP2D6 Activity Score (AS) was first published ten years ago and, since its introduction in 2008, it has been widely accepted in the field of pharmacogenetics. This scoring system facilitates the translation of highly complex *CYP2D6* diplotype data into a patient’s phenotype to guide drug therapy and is at the core of all *CYP2D6* gene/drug pair guidelines issued by the Clinical Pharmacogenetics Implementation Consortium (CPIC). The AS, however, only explains a portion of the variability observed among individuals and ethnicities. In this review, we provide an overview of sources in addition to *CYP2D6* genotype that contribute to the variability in CYP2D6-mediated drug metabolism and discuss other factors, genetic and non-genetic, that likely contribute to the observed variability in CYP2D6 enzymatic activity.

## 1. Introduction

The highly polymorphic nature of the *CYP2D6* gene locus is the most important single factor explaining the wide range of CYP2D6 activity observed between and among populations [1]. Even if genotype analysis is limited to the most common and clinically relevant major haplotypes, the number of diplotypes typically found in any given population or study cohort remains extremely large. In a previous study that tested for single nucleotide polymorphisms (SNPs) identifying 20 allelic variants as well as copy number variants, we identified 51 and 57 different diplotypes among 273 Caucasians and 210 African Americans, respectively, challenging us to develop a ‘system’ to readily interpret genotype/diplotype information by grouping genotypes into the conventional poor (PM), intermediate (IM), extensive (EM) (now referred to as normal (NM) [2]) and ultrarapid (UM) metabolizer phenotype groups. In order to facilitate the translation of genotype into phenotype, we introduced the Activity Score system (AS), which has subsequently become widely accepted in the field since it was first published 10 years ago [3], as evidenced by a growing body of literature utilizing the AS system and its adoption by the Clinical Pharmacogenetics Implementation Consortium (CPIC) for their drug/gene pair recommendations [4,5,6]. Essentially, each allele is assigned a value of 0, 0.5 or 1 categorizing it as no, decreased or normal function, respectively (note that the value of 0.5 does not indicate a 50% reduction in activity, but signals decreased function, i.e., has functional activity somewhere between no function and full function); for alleles with two or more gene copies, the value of the allele is multiplied by the number of gene copies (e.g. a CYP2D6*1x2 gene duplication receives a value of 2 to calculate the AS). The sum of the values of both alleles provides the AS of a genotype. In most populations, this binning system typically leads to six AS groups, i.e., AS = 0, 0.5, 1, 1.5, 2, and ≥3. This “scaled” system is easy to use and more intuitive than “star” diplotypes (for example CYP2D6*29/*58), which are difficult to interpret for many clinically oriented healthcare professionals who are not intimately familiar with CYP2D6 nomenclature and therefore may struggle with assessing the clinical impact of a particular *CYP2D6* genotype.

Although the use of the AS system has simplified genotype–phenotype associations, it has become increasingly clearer over the past 10 years that (1) the approach will benefit from additional refinement, and (2) additional factors contribute to variability in CYP2D6 activity within a given diplotype, and are not captured by the current scoring system. More research in these two areas will potentially allow for more widespread clinical applicability of the AS. In terms of refinement, there currently are more AS groups than the four phenotype classifications, i.e., poor, intermediate, normal (extensive) and ultrarapid that have traditionally been used throughout the literature as well as for clinical test reporting. While there is consensus among experts to define AS = 0 as PM, AS = 0.5 as IM, AS = 1.5 and 2 as NM and those with an AS ≥ 3 as UM, experts are divided regarding the classification of subjects with AS = 1 and 2.5. AS = 1 is the most contested AS group and classified by some investigators as IMs and NMs by others, causing inconsistent group assignment in the literature and may potentially lead to different recommendations by clinically authoritative groups such as the CPIC and the Dutch Pharmacogenetics Working Group (DPWG). Genotypes giving rise to an AS = 2.5 are typically binned with AS ≥ 3 and classified as UM, but some experts suggest that NM may be a more appropriate assignment. Second, the classification of alleles as increased, normal, decreased or no function is crude, and does not take into account substrate-dependent effects of an allelic variant toward various CYP2D6 substrates [7]. This challenge is perhaps best exemplified by CYP2D6*10, a decreased function allele, which has been associated with substantially reduced levels of activity. For example, the kinetic parameters of the CYP2D6.10 protein result in intrinsic clearance values that range from 1.3% to 27.9% of the reference CYP2D6.1 protein for nortriptyline 10-hydroxylation and codeine *O*-demethylation, respectively [7]. Furthermore, the current assignment of a value of 0.5 to this allele classifies homozygous CYP2D6*10/*10 patients as NM in CPIC guidelines, which may not be appropriate for all drugs. A literature review and assessment performed in 2013 [8] did not produce sufficient evidence to recommend a reclassification of the CYP2D6*10 allele by assigning a lower value of 0.25 to better reflect the level of reduction of this allele at that time. However, a recent extensive literature review and assessment performed for the *CYP2D6*/tamoxifen gene/drug pair CPIC guideline produced strong evidence for a separate recommendation for CYP2D6*10-containing genotypes [9], fueling discussions regarding the classification of this particular allele and the translation of CYP2D6*10 diplotypes into phenotype. Another example is CYP2D6*17, which is generally considered to be a decreased function allele, but appears to have normal or even increased activity towards risperidone [10]. In vitro investigations by Shen et al. exploring the impact of CYP2D6*10 and CYP2D6*17 on prototypical substrates (bufuralol, dextromethorphan, debrisoquine, atomoxetine, (*S*)-fluoxetine, nortriptyline, tramadol, and codeine) underscore this point by demonstrating marked substrate-specific and allele-specific metabolism towards these substrates [7]. Specifically, decreases in intrinsic clearance, a measure of enzymatic activity for a given substrate, were observed for all drugs when incubated with either CYP2D6.10 or CYP2D6.17 protein. Wild-type-to-variant intrinsic clearance ratios ranged from 1.32–27.9 and 7.33–80.4 for CYP2D6.10 and CYP2D6.17, respectively. The magnitude of lowered metabolic capability, therefore, appears to be both substrate and genotype dependent [7]. As such, these data illustrate the complexity of translating genotype into phenotype and further highlights the difficulties when using this information in a clinical setting for a given drug. Implicit to this issue is a need to standardize the translation of genotype (or AS) into phenotype, e.g., which panel of substrates used to make this determination, level of evidence needed, etc. The CPIC is currently working with a group of CYP2D6 experts to find consensus for possible solutions.

**Figure 1:**
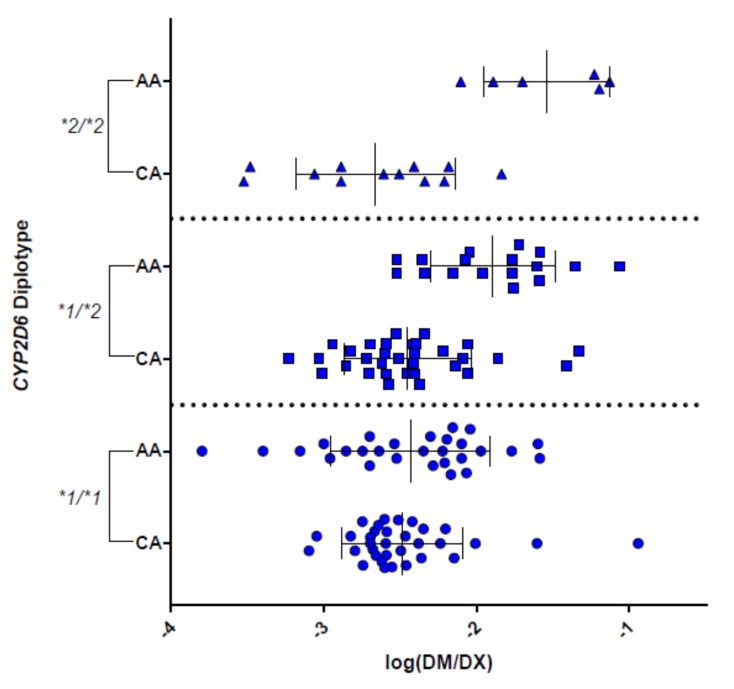
The urinary dextromethorphan/dextrorphan (DM/DX) ratio serves as a measure of CYP2D6 activity. The log-transformed ratios were stratified by *CYP2D6* diplotype (CYP2D6*1/*1, *1/*2, and *2/*2) and ethnicity (Caucasian (CA) vs. African-Americans (AA)). Statistically significant differences in activity were observed between these two ethnic groups for CYP2D6*1/*2 (*p* < 0.0001) and *2/*2 (*p* = 0.0003).

Not addressed by the current AS system is the observation of large intra- and inter-individual variability in CYP2D6 activity within a given genotype group, which remains unexplained. Individuals phenotyped at multiple occasions with the probe drug dextromethorphan (DM) have been shown to present with urinary metabolic ratios that varied between 1.6 and 41-fold [11]. Another study using DM and metoprolol as probe drugs showed inter-individual differences ranging 24–75% and 21–96%, respectively [12]. Regardless of the probe drug, large inter-individual variability within a genotype group is typically observed when urinary metabolic ratios are used as a measure of in vivo CYP2D6 activity [3,13,14,15]. In our own study [3], we also observed a notable difference among Caucasian and African American cohorts when comparing particular genotype groups, namely CYP2D6*1/*1, *1/*2 and *1/*2 suggesting additional source(s) of variability (Figure 1; data from [3]). Further investigation into the interaction of ethnicity with *CYP2D6* genotype–phenotype relationships is warranted given that CYP2D6*1 and *2 are commonly assigned haplotypes in most populations [16]. Considerable variability has also been described in a pharmacokinetic (PK) study investigating the contribution of *CYP2D6* alleles on activity [17]. The authors determined that the 95% confidence interval for the point estimates varied significantly for CYP2D6*1, *2 and *41, the three alleles that were analyzed in that study. As an alternative to urinary metabolic ratios, CYP2D6 activity can also be estimated using plasma or saliva metabolite ratios [18,19,20,21]. Furthermore, data from therapeutic drug monitoring can be utilized as convenient measures to determine CYP2D6 phenotype as demonstrated by Mannheimer et al. [22] and commented on by de Leon [23]. However, regardless of which phenotyping method is employed, large inter-individual differences are observed within genotype groups as well as AS groups, making it difficult to determine a patient’s ‘absolute’ phenotype. 

The goal of this review is to describe the advances that have occurred over the past 10 years to increase our understanding of various genetic and regulatory factors that may contribute to variability in CYP2D6 activity, and thus improve the AS system and prediction of a patient’s CYP2D6 activity.

## 2. Genetic Factors Impacting mRNA and Protein Expression Levels

In addition to variation within the *CYP2D6* gene that causes amino acid changes or leads to alternative splicing, activity towards a particular drug substrate may also be impacted by regulatory mechanisms, including genetic polymorphisms in enhancer regions or differential expression of transcription factors or micro RNAs that can modulate the rate of the translation of mRNA into protein. These mechanisms are typically not ‘all or nothing’, but rather fine-tune level of expression and thereby protein activity. The following sections provide an overview of what is known about these factors.

### 2.1. Long-Range Enhancer Single Nucleotide Polymorphism

To better understand the networks connecting and regulating P450 expression levels and uncover factors controlling these genes, Yang et al. performed comprehensive genome-wide association analyses in a large panel of 466 human liver tissue samples and concluded that both *cis*- and *trans*-regulation are contributing to P450 expression and activity levels [24]. For CYP2D6, their systems biology approach exclusively discovered *cis*-SNPs that were associated with expression (eSNPs) and/or enzymatic activity (aSNPs) laying the groundwork for future investigations looking for genetic variation outside of the coding gene and immediate flanking/regulatory regions. Searching for such regulatory SNPs that might explain the variation of activity within a genotype group, Wang et al. subsequently demonstrated that a SNP (rs16947, R296C) in exon 6 that defines the CYP2D6*2 haplotype not only causes an amino acid change that appears to be of no functional consequence, but causes alternative splicing leading to at least a 2-fold decrease in expression levels [25]. In addition, two distant SNPs located 116 kilo base pairs downstream of the *CYP2D6* gene locus appeared to increase transcription levels over 2-fold. These “enhancer” SNPs were found to be in complete linkage with each other and also in linkage disequilibrium with rs16947, i.e., when rs16947 was present the enhancer SNPs were also present more often than not. In addition to quantifying the amount of alternatively spliced mRNA products in human liver tissue samples to assess the impact of the enhancer SNPs on CYP2D6 activity, Wang et al. also assessed 164 subjects who were phenotyped by our group with the CYP2D6 probe drug DM [25]. Individuals with the enhancer SNPs and rs16947 had less activity (or a higher DM/dextrorphan (DX) ratio) compared to those individuals who had rs16947 and lacked the enhancer SNPs, suggesting that the differences in mRNA expression levels can decrease in vivo activity. These findings led to a series of follow up investigations by the same group, in which the authors further characterized the enhancer element harboring the two SNPs [26]. First, an approach of chromatin conformation capture combined with next-generation sequencing, chromatin immunoprecipitation (ChIP) and reporter gene assays confirmed the distant region as a regulatory element for CYP2D6 expression. Second, deleting selective enhancer region sequences with CRISPR in HepG2 cells identified rs5758550 as the functional SNP. The authors concluded that, taken together, their findings strongly support a functional role of the rs5758550 enhancer SNP that may explain some of the observed unaccounted variability in CYP2D6 activity.

**Figure 2:**
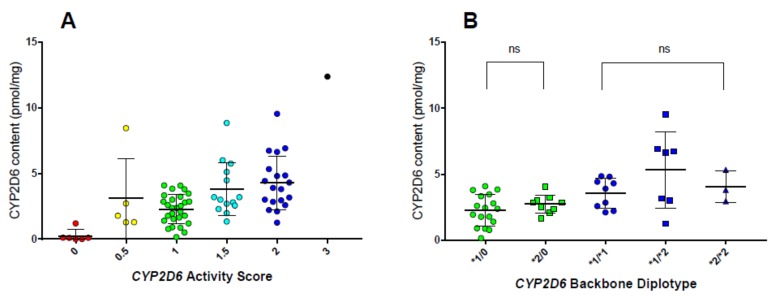
(**A**) CYP2D6 protein content (pmol/mg microsomal protein) stratified by CYP2D6 activity score (AS) in pediatric human liver microsomes (HLMs) (*n* = 78, ages 2–18 years). A statistically significant linear trend was observed with increasing protein content corresponding with increasing CYP2D6 AS. One-way ANOVA analysis identified only significant differences between HLMs of AS = 0 and HLMs with scores greater than 0; (**B**) a subset of pediatric HLMs (*n* = 45) were used to analyze CYP2D6 protein content as a function of activity score (green symbols, AS = 1; blue symbols, AS = 2) and backbone diplotype (allelic variants containing a *1 or *2 structure). A haplotype with an AS = 0 indicates the presence of two *CYP2D6* allelic variant with no functional activity. No statistically significant changes in protein content were observed as a result of differing activity score or backbone diplotype.

The presence or absence of the enhancer SNP may explain, at least in part, the wide range of activity among individuals (Figure 1) or tissue samples (Figure 2) genotyped as CYP2D6*1/*2 or *2/*2 for example, or why some individuals genotyped as CYP2D6*1/*1 or *1/*2 may have activity resembling that of an ultrarapid metabolizer. Wang et al. proposed to reclassify alleles based on the combination of rs1694 (2850C>T) and rs5758550 as following: rs16947T/rs5758550A (reduced activity), rs16947T/rs5758550G (normal activity) and rs16947C/rs5758550G (enhanced activity) [26]. Whether this approach is superior over the AS System or other classification system remains to be shown. Of note, the enhancer SNP is not in complete linkage with rs1694 (2850C>T); it can occur on haplotypes that do not have rs1694 (2850C>T) including CYP2D6*1, *5 and possibly *10, and also appears to be present on a portion of decreased function alleles with rs1694 (unpublished data). In other words, if a patient is genotyped as CYP2D6*2/*5 and is heterozygous for the enhancer SNP, it is impossible to determine whether the patient has a decreased function CYP2D6*2 allele (rs16947T/rs5758550A) or a normal function CYP2D6*2 allele (rs16947T/rs5758550G) according to the classification system proposed by Wang et al. [26]. Furthermore, there is no information regarding the impact of the enhancer SNP on the activity for other haplotypes including CYP2D6*41, which is currently classified as a decreased function allele. Finally, little is known regarding the frequency of the enhancer SNP in other racial or ethnic populations and the linkage of the enhancer SNP with defined haplotypes.

The enhancer SNP certainly holds promise to explain inter-individual variability in CYP2D6 activity and eventually be incorporated into *CYP2D6* genetic testing. However, more information is needed on the identity of star alleles that are linked with the enhancer SNP, their function (with and without the enhancer SNP), and their frequency distributions across populations. Lastly, for heterozygous patients, methods need to be developed to determine on which allele the enhancer SNP is located to interpret the result and more accurately predict the patient’s phenotype compared to current practice.

### 2.2. Regulation of CYP2D6 Expression via Transcription Factors

For the last two decades, studies have shown that expression of most drug-metabolizing enzymes (e.g., CYP3A4) is inducible upon activation of transcription factors such as pregnane X receptor (PXR) or constitutive androstane receptor (CAR) [27,28]. PXR and CAR are ligand-activated nuclear receptors to which various xenobiotics bind, and considered to serve as xeno-sensors for the bodies to promote elimination of foreign compounds. Of interest, these transcription factors failed to transactivate the promoter of *CYP2D6* although CYP2D6 is one of the major drug-metabolizing enzymes (mediating metabolism of >20% of marketed drugs). This has led the research community to regard *CYP2D6* as a non-inducible gene. Challenging this notion, however, accumulating clinical evidence has indicated increased CYP2D6-mediated drug metabolism (e.g., higher clearances or metabolites/parent drug ratios) in pregnant women as compared to postpartum women [29,30,31,32]. The underlying mechanisms were unknown, in part due to a lack of appropriate models (e.g., animals or in vitro cell systems) that could recapitulate the clinical phenotype. A breakthrough was made in a recent study where pregnancy-mediated CYP2D6 induction was observed in CYP2D6-humanized mice, a transgenic mouse line whose genome harbors the human *CYP2D6* gene along with its upstream gene-regulatory region [33]. In these mice, CYP2D6 mRNA level increased about 3-fold at term pregnancy (as compared to pre-pregnancy or postpartum), subsequently leading to higher CYP2D6 enzyme activity [33]. Importantly, the results strongly suggest the roles of transcriptional regulation in CYP2D6 induction during pregnancy.

The CYP2D6 promoter was first characterized in the late 1990s [34], where promoter reporter assays revealed a proximal DNA sequence in the *CYP2D6* promoter that binds to a transcription factor, hepatocyte nuclear factor 4α (HNF4α). HNF4α is a nuclear receptor highly expressed in the liver, regulating constitutive expression of many liver-specific genes (including ones involved in nutrient metabolism and blood coagulation) [35]. Important roles of HNF4α in the regulation of basal CYP2D6 expression was further verified in a study where a rare polymorphism in *HNF4α* (i.e., G60D; minor allele frequency of 1.3% in Koreans) that causes decreased HNF4α binding to a *CYP2D6* promoter was shown to be associated with lower CYP2D6 expression and activity in liver tissues [36]. In CYP2D6-humanized mice, however, hepatic HNF4α expression (at both mRNA and protein levels) did not differ among different gestational time points [33], suggesting that altered hepatic expression of HNF4α is not responsible for CYP2D6 induction in the pregnant mice. Of note, multiple transcription factors are known to modulate HNF4α activity via physical interactions [35]. cDNA microarray experiments of mouse liver tissues revealed eight transcription factors that are differentially expressed at term pregnancy [33,37]. Transient transfection and promoter reporter assays in cell lines revealed that among the eight transcription factors, Krüppel-like factor 9 (KLF9; upregulated at term pregnancy) and small heterodimer partner (SHP; downregulated at term pregnancy) are capable of modulating HNF4α transactivation of the *CYP2D6* promoter. Specifically, KLF9 enhanced HNF4α action on the *CYP2D6* promoter while SHP repressed it. Hepatic delivery of siRNA against SHP in nonpregnant mice led to enhanced CYP2D6 expression, verifying the repressive role of SHP in the regulation of basal CYP2D6 expression in vivo [33]. Extensive search for upstream regulators of KLF9 and SHP expression during pregnancy led to the following finding: hepatic levels of retinoids that are known inducers of SHP expression [38] are reduced at term pregnancy in CYP2D6-humanized mice [33]. Intraperitoneal administration of all-trans retinoic acid (atRA; the bioactive retinoid) to nonpregnant humanized CYP2D6 mice led to increased SHP and decreased CYP2D6 expression by about 2-fold, indicating that retinoids are indeed capable of regulating hepatic CYP2D6 expression [33].

Expression of SHP is known to be modulated by multiple factors including drugs or diseases (e.g., cholestasis). SHP is a representative target gene of the farnesoid X receptor (FXR), a bile acid sensor. In cholestasis, hepatic bile acids bind to and activate FXR, leading to transactivation of the SHP promoter [39]. SHP in turn represses expression of genes encoding enzymes for bile acid synthesis [40]. A synthetic agonist of FXR, GW4064, decreased CYP2D6 expression in human hepatocytes as well as in CYP2D6-humanized mice [41]. Ethinylestradiol, a female hormone that is known to cause cholestasis when administered at a high dose, also repressed hepatic CYP2D6 expression in CYP2D6-humanized mice [42]. Together, these results suggest that multiple factors may govern basal hepatic CYP2D6 expression via regulation of CYP2D6 transcription, and this may be in part responsible for inter-individual variability in CYP2D6-mediated drug metabolism. Supporting the notion, previous studies have shown that CYP2D6 activities well correlate with its mRNA expression levels in human liver tissues [24,43,44,45]. However, the extent of the contribution by differential CYP2D6 transcription to overall variability in CYP2D6 activity remains to be defined.

### 2.3. Regulation of CYP2D6 Expression via miRNA

An increasing body of evidence demonstrates that small, noncoding RNAs, ranging between 20 and 24 nucleotides in length, provide an additional layer of regulation on the expression of drug metabolizing enzymes including CYPs [46,47,48] and that sequence variation in miRNA binding sites can perturb this mechanism as exemplified in vitro and in vivo by Burgess et al. for the regulation of CYP2B6 expression [49]. Consistent with the finding that miRNA binding sites are often located in the 3’UTR region, the most prominent examples of CYP regulation by miRNAs involve binding sites in this region [49,50,51,52,53]. A bioinformatic search using 15 databases revealed that the 75 bp long 3’UTR of *CYP2D6* contains a number of potential miRNA binding sites [47]; however, the author also indicated that this relatively small region does not harbor any known sequence variations that may interfere miRNA-mediated regulation. More recently, Zeng et al. reported that the *CYP2D6* 3′UTR does not contain any putative miRNA binding sites using four databases, but their search revealed that the coding region of the transcript may be targeted by several miRNAs, among them hsa-370-3p [54]. A potential role of this miRNA was supported by a negative correlation of hsa-370-3p with hepatic CYP2D6 mRNA expression levels. In a subsequent series of experiments, Zeng et al. provided strong evidence that this miRNA indeed impacts CYP2D6 expression. First, using RNA electrophoretic mobility shift assays, a direct interaction between hsa-miR-370-3p and the CYP2D6 mRNA was demonstrated. Next, Zeng et al. performed a series of cell culture experiments showing that the expression of exogenous CYP2D6 mRNA and protein was suppressed by hsa-miR-370-3p and that hsa-370-3p is capable of attenuating the induction of CYP2D6 both on the mRNA and proteins levels. Furthermore, Zeng et al. demonstrated that hsa-miR-370-3p decreases the stability of CYP2D6 mRNA by significantly shortening the half-life of the transcript. Taken together, Zeng et al. provided a strong body of evidence that CYP2D6 expression undergoes regulation by miRNA, albeit not by targeting the 3′-UTR.

The region targeted by hsa-370-3p corresponds to cDNA + 1296–1317 and maps to exon 8 and one nucleotide of exon 9. There are no known validated sequence variations in this gene region that may directly impact miRNA-mediated regulation. There are no studies that the authors are aware of that have evaluated hsa-370-3p, mRNA and protein expression levels within a given genotype group to assess whether hsa-370-3p levels contribute to the range of CYP2D6 activity observed among a genotype group. It remains to be seen whether other miRNAs emerge in the future that modulate CYP2D6 activity either directly or indirectly. 

### 2.4. Variability in the Amount of CYP2D6 Protein among Liver Tissue Samples with the Same Activity Score or Genotype

Genetic variants can influence both the abundance of enzyme expressed as well as the catalytic activity of the expressed variant isoform. To date, over 100 *CYP2D6* allelic variants and subvariants have been defined by the Human Cytochrome P450 Nomenclature Committee, which has recently transitioned to the Pharmacogene Variation Consortium (PharmVar) at [55]. The vast majority of allelic variants in the database occur within the coding region of the gene and harbor detrimental sequence variations, such as single nucleotide deletions or insertions causing a frameshift and leading to premature termination characteristic of CYP2D6*3, *6 or *15. Similarly, aberrant splicing due to a SNP at a splice site, such as those in CYP2D6*4 and *11, can also lead to the absence of functional protein rendering the allele nonfunctional. Allelic variants associated with impaired capacity to metabolize substrates do so either through (1) a decrease in functional protein content through alternative splicing or gene deletion events or (2) altered binding affinity between enzyme and substrate caused by amino acid changes affecting substrate–enzyme interactions. Lower active protein content without a change in enzyme catalytic efficiency is characteristic of CYP2D6*9, *10 and CYP2D6*41 [56,57,58].

Preliminary data indicate that microsomal CYP2D6 protein content varies within livers with nominally identical diplotypes, suggesting that identification of genetic and non-genetic factors influencing protein expression may provide an opportunity for refining the AS system. Using a collection of pediatric human liver microsomes (HLMs), protein abundance determined by liquid chromatography with tandem mass spectrometry-based proteomics (ages 2–18, *n* = 73) was positively associated with AS, supporting the existence of a gene dose–protein content relationship, where on average the CYP2D6 protein content increases with increasing AS. There is, however, considerable variability in protein content among samples with the same AS such that there is extensive overlap of protein contents between AS groups (Figure 2A), similar to the extensive overlap in log(DM/DX) urinary metabolite ratios between AS groups (Figure 1). The extent of variability in protein content within AS groups ranges between 6- and 22-fold, with HLMs with an AS = 1 having the greatest extent of variation (only HLMs with AS ≥ 0.5 and AS groups with ≥2 samples included in fold-variability calculations). This latter observation may be explained by the fact that this group comprises diplotypes consisting of one functional and one nonfunctional allele, as well diplotypes comprised of two decreased function alleles. Of particular note, within individual livers with the same *CYP2D6* diplotype, marked variability in CYP2D6 abundance is still observed (Figure 2B). Using atomoxetine as a CYP2D6 substrate, catalytic activity was associated with protein content, offering one potential explanation for variability in atomoxetine clearance within AS groups [59]. Especially intriguing is the possibility that there may be distinct groups based on proteomic determination of CYP2D6 protein content in livers genotyped as CYP2D6*1/*1 and *1/*2. These data suggest that other factors, such as long-range regulatory SNPs such as the distant enhancer SNP described above, may contribute to variability in CYP2D6 protein expression. Ning et al. investigated a panel of 115 adult human liver tissues showing that protein content is a better predictor of CYP2D6-mediated drug metabolism compared to AS (23% of the variability of CYP2D6 activity was explained by AS while protein content explained 59%) [60]. Further work is, however, required to verify their and our findings in a larger number of samples, and to elucidate the mechanisms leading to the variability in protein abundance within genotypes. Identification of the factors involved will allow continued refinement of the AS, and ultimately better prediction of drug clearance, and thereby individual dose requirements for drugs cleared by the CYP2D6 pathway. 

### 2.5. Missing Genetic Information: Variability Due to Untested Variation, Variation of Unknown Function, Novel Variants and Technical Errors

Genotype panels typically test for a limited number of SNPs identifying more commonly observed *CYP2D6* alleles and provide limited information on gene copy number and structural variants. It is therefore always possible that a patient carries an allele(s) that was not tested for. For example, a number of no function alleles including CYP2D6*11 and *12 are genotyped as CYP2D6*2 if not tested. Likewise, if CYP2D6*15 or *44 are not tested, these alleles are reported as CYP2D6*1. Because CYP2D6*1 and *2 are ‘default’ assignments, it is not inconceivable that the CYP2D6*1/*1, *1/*2 and *2/*2 genotype groups may contain individuals with rare, untested alleles that contribute to the variability seen in these groups. 

Although numerous sequence variations have been described for *CYP2D6* and have been defined by PharmVar, novel variants continue to be discovered, especially in ethnic populations that have not been well characterized in the past [14,61,62]. Furthermore, novel variants and haplotypes are also being discovered in the major ethnic populations using next-generation sequencing [63,64]. It is therefore always a possibility that a subject carries a novel, not yet discovered variants that contributes to altered metabolism. There are also a number of variants for which there is no or only limited information regarding activity, and the majority of those are not routinely tested and are likely ‘defaulted’ to CYP2D6*1 or *2 unless other SNPs are detected.

Finally, there is a growing number of reports describing genotype errors due to the presence of rare or unknown SNPs, or SNPs within the amplified gene region that interfere with some TaqMan assays (Thermo Fisher Scientific, Waltham, MA, USA) [65,66,67] (some, but not all of these assays have been redesigned by the manufacturer to avoid such errors). Depending on the platform used and the nature of the second allele, an allele conferring decreased or no function may be missed altogether or may be determined to be homozygous, leading to inaccurate phenotype assignments. Other platforms may produce ‘no-calls’ due to inconsistent SNP patterns [68,69] as reported for the AmpliChip CYP450 Test (Roche Molecular Diagnostics, Alameda, CA, USA, a product that is no longer available.

## 3. Other Factors That May Modulate CYP2D6 Expression Levels or Enzyme Activity

It must be emphasized that the AS is simply a starting point for predicting CYP2D6 phenotype, and that a multitude of individual-specific factors, such as environment or co-administration of CYP2D6 substrates or inhibitors, may affect the actual phenotype, or activity, of a person at a given time. The following sections are not intended to be all-inclusive, but rather exemplify why some individuals may present with activity at the extreme ends of the distribution within their genotype group, or with a phenotype that is discordant with the metabolizer status predicted by his/her genotype. Use of the AS to guide clinical decision-making should also consider the factors described below, amongst other patient factors.

### 3.1. Competing Pathways

Compounds used as phenotyping probes are principally metabolized by the CYP of interest, such as DM, debrisoquine and sparteine for CYP2D6. The ideal substrate to be used as a phenotyping probe, the pathway of interest should be exclusively responsible for the clearance of the probe [70]. In reality, however, almost every CYP substrate is subject to biotransformation and clearance by multiple competing pathways, including those used as phenotyping probes. For example, dextrorphan can be detected in urine from CYP2D6 PMs, which is consistent with the observations that CYPs other than CYP2D6 catalyze *O*-demethylation of DM [71]. Additionally, DM also undergoes *N*-demethylation by other CYPs, such as CYP3A4 and CYP2C19 [71]. The impact of competing pathways varies depending on the percent contribution of each enzyme on the metabolism and clearance of the parent drug. Thus the magnitude of dose adjustments based on CYP2D6 AS or predicted phenotype (PM, IM, NM, UM) will be drug-dependent, as reviewed by Stingl et al. [72]. Furthermore, the relative contribution of pathways may shift in the presence of an inducer. CYP3A4 for instance may be a minor pathway under normal circumstances, but may become more prominent when a CYP3A4-inducing agent is co-administered [73]. Alternative pathways may also play a more prominent role in CYP2D6 poor metabolizers compensating for the absence of CYP2D6 activity. Essentially, differences in the relative contribution of CYP2D6 relative to the sum of all other competing pathways to overall drug clearance is reflected by the magnitude of the difference in clearance or systemic drug exposure between PMs and NMs (generally with two fully functional alleles).

### 3.2. Drug–Drug Interactions

The PK profile of a drug may be substantially altered in the presence of an inhibiting agent. Numerous drugs including antidepressants (e.g., paroxetine and fluoxetine) are known to interact with CYP2D6. Such drugs can cause phenoconversion meaning that an individual can presents with lower or no activity compared to that predicted by a genotype test due to inhibition of the CYP2D6 enzyme. For a detailed review of drug–drug-interactions (DDIs) involving CYP enzymes, we recommend readers to refer to Bahar et al. [74].

Although study participants are typically asked to provide information on all medications taken, there is always the possibility that not all are disclosed. Such cases do not necessarily present as phenotype–genotype discordant, but may add to the variability observed among individuals of the same genotype. The degree of inhibition depends on the potency of the inhibitor, but also the particular allelic variants composing a patient’s genotype. Investigating DDIs between duloxetine, paroxetine, dextromethorphan and tramadol, Storelli et al. [75] show that individuals with one functional allele (AS = 1) converted to poor metabolism at a higher rate compared to those with two functional alleles (AS = 2). The authors conclude that future research needs to investigate additional genetic variants not tested by their study to better predict DDIs. They also discuss that physiologically-based pharmacokinetic (PBPK) modeling, taking all known factors into account including genetics, concomitant medications, etc. that contribute to a patient’s metabolic profile, may be the most promising approach to better predict a patient’s activity at a given time.

### 3.3. Herbal Remedies

As reviewed by Thomford et al. [76], herbal remedies and supplements are generally considered safe by most people and not considered to be ‘drugs’, but ‘natural’ and therefore harmless. Supplements may be taken for preventive and therapeutic purposes including the treatment of the same condition for which conventional drug treatment is sought. Components of herbals may be metabolized through cytochrome P450 including CYP2D6, or act as inhibitors thereby interfering with the metabolism of the prescribed therapeutics or a probe drug administered for phenotyping purposes. There are a number of herbals that are commonly used in West and Southern Africa that have been reported to interact with CYP2D6. *Hyptis suaveolens*, also known as bush mint, bush tea or pignut, for example, is found in many African countries, but also other tropical and subtropical regions around the works including China, South America and the United States. Bush mint is used to treat a wide range of diseases and ailments including cancer, diabetes, malaria, hepatitis and eczema, to name a few. Thomford et al. [77] have shown in a recent report that crude bush mint extracts inhibit CYPs 1A2, 3A4 and 2D6, with the strongest inhibition observed towards CYP2D6. Such herbal–drug interactions have the potential to adversely interfere with drug therapy.

### 3.4. Physiological Factors That May Impact CP2D6 Expression and Activity

In addition to DDIs, there is growing evidence that biological and physicochemical factors may also influence phenotype prediction from AS. A growing body of evidence indicates that certain pro-inflammatory cytokines are released during inflammatory processes and can act as modulators of *CYP* gene expression [78,79] directly or indirectly affecting CYP2D6 activity. Inflammation is a complex protective response that can be triggered by a range of stimuli and present as acute (infections) or chronic (e.g. allergic reactions, diabetes, asthma). The “Impact of physiological, pathological and environmental factors on the expression and activity of CYP2D6 and implications in precision medicine” has extensively been reviewed by He et al. [80]. Patients with hepatitis C, for instance, had lower CYP2D6 activity compared to those not infected. On the other hand, diabetes and rheumatoid arthritis do not appear to impact CYP2D6 activity. Although participants in phenotyping studies are generally described as ‘healthy’, extrinsic factors such as an infections or other intermittent processes could conceivably modulate drug metabolism and contribute to intra-individual variability, and thus cause a person to deviate considerably from the mean activity observed for a genotype. Inflammation may also exert an affect by impacting a CYP or other pharmacogene shifting the relative contribution of CYP2D6. Clearly, more research is needed to more fully understand the extrinsic factors that play a role in CYP2D6 variability.

In addition to inherent variability in renal excretion, urinary pH can also impact the apparent urinary metabolic ratio and contribute to variability within a genotype group. Labbe et al. have shown that a large portion (up to 80%) of variability observed in urinary ratios for the probe drugs dextromethorphan and metoprolol can be explained by variation in the urinary pH within the physiological range [12]. Hence, urinary pH should be accounted for when using urinary metabolic ratios. 

Furthermore, we are only starting to gain insights into the contribution of the microbiome on overall health and its role in drug metabolism and response [81]. A recent study also provided evidence that nutritional status, specifically fasting, alters P450-mediated drug metabolism in mice [82]. Whether, and to what extent, a person’s microbiome, nutritional and fasting status influences CYP activity and CYP2D6 metabolizer status, intra- and interindividual variability, in particular, remains to be explored, however.

## 4. Conclusions

Over the past 10 years, the concept of ‘activity score’ has gained acceptance as a tool to easily predict CYP2D6 phenotype from a reported genotype/diplotype, and has been adopted for CPIC guidelines intended to facilitate the application of pharmacogenetics knowledge into clinical care. Nevertheless, it remains a crude tool, as considerable inter-individual variability in drug clearance and other measures of phenotype exist within a given AS group. Characterization of the genetic factors contributing to intra-activity score variability may allow refinement of the AS system, and identification of non-genetic factors may be included as covariates in decision support tools based on AS to individualize dosing of CYP2D6 substrates.
